# Platelet-Rich Plasma Versus Saline for the Treatment of Vulvar Lichen Sclerosus: Protocol for a Randomized Controlled Trial

**DOI:** 10.2196/68871

**Published:** 2025-09-03

**Authors:** Tran Nguyen, Milica Beljic, Nadia Willison, Mahshid Taheri, Pouria Aryan, Derek Abbott, Fariba Behnia-Willison

**Affiliations:** 1 Discipline of Biomedical Engineering School of Electrical and Mechanical Engineering University of Adelaide Adelaide Australia; 2 FBW Gynaecology Plus Ashford Australia; 3 School of Medicine and Public Health Flinders University Adelaide Australia

**Keywords:** regenerative medicine, vulvar dermatoses, female sexual health, autologous therapies, clinical trial protocols, women’s health research

## Abstract

**Background:**

Vulvar lichen sclerosus (LS) is a chronic relapsing dermatosis commonly affecting the anogenital region in postmenopausal women, though it can affect people of any age and sex. The current gold standard treatment is lifelong topical steroid application to reduce symptoms and prevent the progression of disease, causing irreversible architectural change to the vulval tissue. LS is associated with decreased quality of life and increased risk of vulvar neoplasia. Alternatives to current treatments are highly desired by both clinicians and patients. Platelet-rich plasma (PRP) is an autologous blood product containing high concentrations of platelets and growth factors and is hypothesized to promote wound healing. PRP has been reported to improve symptoms of LS in several case reports and uncontrolled trials.

**Objective:**

This study aims to evaluate the efficacy of PRP versus saline injections to improve the clinical signs and symptoms of LS.

**Methods:**

This is a protocol for a 12-month, single-center, parallel-group, double-blind, randomized controlled trial evaluating the efficacy of PRP for LS compared to saline. Adult female patients with biopsy-proven LS who are otherwise in good health but are nonresponders to topical steroid treatment prior to inclusion are eligible for the trial. A total of 68 participants will be randomly allocated via a random number generator to receive either PRP or saline injections to areas of the vulva with LS. All participants will be blinded to the intervention received. Participants will be followed up with assessments of clinical LS signs, subjective symptom scores, and quality of life questionnaires by blinded assessors at 4 timepoints: baseline, 6 weeks, 6 months, and 12 months. The primary outcome is the clinical scoring system for LS—the total LS score.

**Results:**

Recruitment commenced in May 2020 and concluded in November 2023. This study closed in September 2024 due to slow recruitment. Data are being analyzed in 2025, and results are expected to be published in late 2025.

**Conclusions:**

This study will evaluate the safety and efficacy of PRP injections compared to those of saline injections for the treatment of vulvar LS, potentially providing a novel therapeutic option for patients who do not respond to topical steroids.

**Trial Registration:**

Australia and New Zealand Clinical Trials Registry ACTRN12618001321235p; Universal Trial Number: U1111-1207-4893; anzctr.org.au/Trial/Registration/TrialReview.aspx?id=375536

**International Registered Report Identifier (IRRID):**

DERR1-10.2196/68871

## Introduction

### Background

Vulvar lichen sclerosus (LS) is a chronic autoimmune dermatosis with a complex and multifactorial etiology [1,2]. In females, LS typically affects the anogenital region, including the vulva, perineum, and anus, but extragenital involvement occurs in 6%-20% of the patients [3,4]. The characteristic signs of LS are patches of discolored white skin, and the typical symptoms are intense itching, burning, and pain [5-7], which can contribute to significant distress and discomfort. Over time, chronic changes to the architecture of the vulva can occur, such as agglutination of the labia and narrowing of the introitus [3]. Additionally, LS is associated with an increased risk of vulval intraepithelial neoplasia and vulvar squamous cell carcinoma, with an estimated malignant transformation risk of approximately 4% to 6% [8,9]. The incidence of LS has been estimated at 22 per 100,000 woman-years in population studies [10], and it accounts for about 1.7% of the referrals to general gynecology clinics [11,12].

There is no cure for LS, and the current mainstay of therapy is topical corticosteroid application [3]. The goals of the therapy are symptom relief and improving the quality of life, preventing the progression of disease to alter the architecture of the skin, and reducing the risk of neoplasia [3].

Platelet-rich plasma (PRP) is an autologous platelet concentrate, which contains the platelet-derived growth factor, transforming growth factor, and epidermal growth factor, and it is hypothesized to promote wound healing and tissue regeneration [13,14]. It has yielded positive results in orthopedic, reconstructive surgery, dentistry, dermatology, and musculoskeletal medicine [15-18]. PRP has been studied as a treatment for LS [19], but issues with small sample sizes, heterogenous study methodologies, short-term follow-up, and the use of unvalidated outcome measures are the limitations that have prevented the recommendation of PRP use in clinical practice [13]. In the context of LS, PRP may exert therapeutic effects through its anti-inflammatory and immunomodulatory properties. Growth factors such as transforming growth factor-β and platelet-derived growth factor found in PRP can modulate the immune responses and promote tissue remodeling. These effects may reduce local inflammation, improve dermal regeneration, and help restore the structural integrity of vulvar tissues affected by LS. Therefore, this trial has been designed as an adequately powered, placebo-controlled randomized study to evaluate the safety and efficacy of PRP in LS by using validated outcome measures.

In contrast to prior studies [20,21], this trial addresses these limitations by employing a randomized, double-blind, placebo-controlled design with an adequately powered sample size. It also utilizes validated clinical and patient-reported outcome measures, including the clinical scoring system for LS, Dermatology Life Quality Index, and Australian Pelvic Floor Questionnaire, with a planned 12-month follow-up. These methodological strengths are designed to provide robust evidence on the efficacy and safety of PRP for LS.

### Study Aims

The goal of this study is to assess the safety and efficacy of autologous PRP injections for the treatment of vulvar LS compared to saline injections.

## Methods

### Study Design

This is a double-blind, parallel-group, placebo-controlled, randomized trial. The purpose of the trial is to evaluate the efficacy of PRP as a treatment for vulvar LS compared to saline. The trial is conducted at a single center—FBW Gynecology Plus in Adelaide, Australia.

### Study Population

Adult female patients formally diagnosed with LS are the target population for this study.

#### Inclusion Criteria

Patients will be eligible if they remain symptomatic from vulvar LS despite topical steroid treatment or if they are intolerant of or unable to use topical steroids.

#### Exclusion Criteria

The exclusion criteria were as follows: (1) patients on antiestrogens, (2) patients on systemic immunosuppressants such as prednisolone, methotrexate, or biologics that were taken within 12 weeks of commencing the trial, (3) patients with gynecological or breast cancers, (4) patients with autoimmune disorders who require antiplatelet medication, except Sjogren syndrome and LS, (5) patients who are immunocompromised or who have uncontrolled malignant disease, (6) patients diagnosed with lichen planus, psoriasis, candidiasis, vulvar intraepithelial neoplasia, or vulvar carcinoma, (7) patients with vulvodynia, (8) patients with acute vaginal infection or systemic infection, (9) patients on antiplatelet medications, including aspirin, (10) patients without capacity to give consent, and (11) patients who are uncooperative, known to miss appointments, are unlikely to be compliant, or unable to attend regular scheduled visits.

#### Recruitment

Recruitment will be conducted over a minimum of 18 months to allow for adequate enrollment and potential participant withdrawal. Eligible patients presenting to the clinic with a confirmed clinical diagnosis of LS during routine consultations will be systematically screened for study inclusion according to the predefined eligibility criteria. All eligible patients will be informed about the study and invited to participate in a standardized manner (Multimedia Appendix 1). Patients who express interest will have an appointment scheduled with a clinical investigator who is a gynecologist to assess their eligibility and confirm their enrollment. The purpose of this appointment is to obtain consent from the patient, record demographic data, schedule all treatment and assessment appointments, and assign the patient with a unique study identifier number.

#### Withdrawal

Participants who do not complete the full treatment regimen and/or do not complete all assessments will be withdrawn from the study to ensure the quality of data analysis. The reasons for the withdrawal will be documented if provided. Any participant who withdraws from the study will be advised to consult with their referring physician to continue conventional treatment for their LS.

#### Randomization and Blinding

Participants will be allocated to intervention and control groups in a 1:1 allocation ratio. Block randomization will be conducted in random block sizes (4:4 and 2:2) to minimize selection bias. Randomization will be performed using a random number generator to assign a value to a corresponding unique study identifier, which will then be sorted in an ascending order to randomize participants to either group. A randomization list will be stored in a concealed location, password protected, and inaccessible to clinical investigators. Sequentially numbered, opaque, sealed envelopes containing the allocation will be prepared according to the randomization list by a biostatistician who is not involved in the clinical trial. The clinical investigator will administer the study treatments for every participant and will therefore be unblinded to the intervention received. Assessment and scoring of LS will be performed by a coinvestigator. Coinvestigators, data collectors, and participants will be blinded to group allocation until completion of the trial.

To minimize potential performance bias due to the clinician being unblinded, all injections follow a strict, standardized protocol regarding injection volume, anatomical injection sites, needle gauge, and injection technique. The injection process is identical for both PRP and saline groups, with a consistent approach to anesthesia, injection sites, and postprocedural care, as outlined in the study protocol (Multimedia Appendix 2).

### Ethical Considerations

This study has received ethics approval from Bellberry Human Research Ethics Committee (2017-07-546-PRE-1, PRP4LS) on October 2, 2018. All participants provided written informed consent prior to enrolment, as documented in the participant information sheet and consent form (Multimedia Appendix 1). The trial will be conducted in accordance with the National Health and Medical Research Council National Statement on Ethical Conduct in Human Research (2023) incorporating all updates [1]. Participants were informed that their participation was voluntary, that they could withdraw from the study at any time without any impact on their ongoing medical care, and that they could choose whether their data collected up to the point of withdrawal could be retained or removed from the study dataset. All data were deidentified prior to analysis. Participant identifiers were replaced with unique study codes, and no information that could directly identify participants was included in the research database. Participant details were stored in an electronic medical record system, and the deidentified study dataset was kept in a password-protected Excel file located on a secure server at the study site. Only authorized study personnel had access to identifiable data, and all records were handled in accordance with applicable privacy and data protection regulations. No monetary payment was provided to participants for their involvement in the study. Participants were required to arrange their own travel to appointments; however, travel vouchers were available upon request. Findings from this trial will be reported in accordance with the CONSORT (Consolidated Standards of Reporting Trials) statement and disseminated to the scientific community via peer-reviewed journals, conferences, medical education sessions, and patient information sessions.

### Study Intervention

#### Overview

Once enrolled, the trial will require 12 months of participants’ time across 4 appointments.

1. Baseline assessment + treatment 1
2. 6-week assessment + treatment 2
3. 6-month assessment
4. 12-month assessment

**Figure 1:**
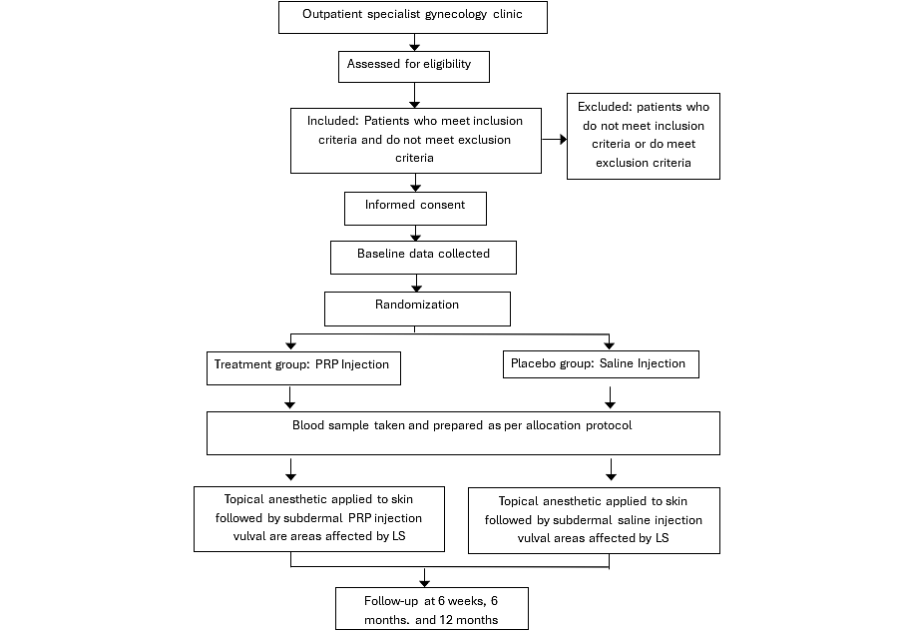
Patient flow diagram. LS: lichen sclerosus; PRP: platelet-rich plasma.

Study treatments can be administered on the same day as assessments take place. Regardless of group allocation, all participants will undergo a blood collection by a clinical nurse who will then access the randomization list and prepare either PRP or normal saline for vulval injection (Figure 1). The patient will have a physical screen to prevent them from seeing the injection to maintain participant blinding.

#### PRP Protocol

At both treatment appointments, 8-10 mL of autologous blood will be collected from the participant into a PRP tube by using the standard phlebotomy technique (Multimedia Appendix 2). The sample is inverted several times to homogenize with the anticoagulant inside the tube. The tube is placed in a centrifuge and set to spin at 3400 rpm for 5-9 minutes. After centrifugation, approximately 4 mL of the concentrated PRP is transferred into a syringe for injection into the vulva.

#### Saline Protocol

The protocol for saline preparation is identical to the PRP protocol except that once the blood is collected into the PRP tube, it is taken out of the consultation room and discarded, and a syringe containing 4 mL of normal saline is drawn up for injection into the vulva.

#### Injection Technique

The participant will be asked to lie in the lithotomy position on the examination table, and a topical anesthetic cream will be applied to the vulva 10-20 minutes prior to injection. The clinical nurse will hand the syringe to the clinical investigator who will inject the study preparation into the vulval mucosa in areas affected by LS by using a 30G needle. The amount injected may be less than 4 mL, if on visual inspection, it becomes evident that the tissue cannot hold the maximum amount.

The decision to administer 2 treatment sessions, spaced 6 weeks apart, was based on protocols from previous PRP studies [20,21] in dermatology and gynecology, where 2-3 sessions are commonly used to optimize tissue response and healing. The 6-week interval allows sufficient time for tissue remodeling and the biological effects of growth factors to occur between treatments. The dosage of 4 mL was selected based on the maximum safe and practical volume deliverable to the vulvar tissue, as well as the prior clinical experience in similar interventions to avoid tissue overdistension or adverse effects. The patient will be monitored for adverse effects for 15 minutes after the study treatment and will be advised not to engage in penetrative vaginal sex for 72 hours after the procedure.

### Data Collection

Each participant is allocated a folder containing the following documents (See Multimedia Appendix 1 and Multimedia Appendices 3-8 for example documents) (Textbox 1).

**Documents in the folder of each participant.**

1. Unique study identifier
2. Sealed opaque envelope containing treatment allocation
3. Trial information sheet and consent form
4. Appointment schedule
5. Baseline demographics sheet
6. Outcomes Assessment Package (4 copies of each):
  Visual Analogue Scale for pruritus and dyspareunia
  Clinical Scoring System for lichen sclerosus
  Australian Pelvic Floor Questionnaire
  Dermatology Life Quality Index Questionnaire
  Study recording sheet

#### Enrollment Appointment

At enrollment, participants receive a trial information sheet outlining the purpose and aims of the trial, intervention details, duration, and participant expectations. The participant is given an opportunity to ask questions before signing a consent form. A copy of the signed consent form is scanned into their electronic medical record. A self-administered baseline demographic sheet is filled out by the participant, which has the following demographic data: name, date of birth, parity, number of children, types of delivery, menopausal status, height, weight, date diagnosed with LS, and the approximate date that the symptoms of LS were first noted (Multimedia Appendix 9).

#### Treatment Appointments

Two treatment appointments are scheduled: at baseline and 6 weeks later. After administering the treatment, the following data are recorded on the treatment record form: date, participant name and date of birth, unique study identifier, verification of venous blood collection, 2-person verification of treatment syringe contents, 2-person verification of injection of treatment syringe, and adverse reactions.

#### Assessment Appointments

Four assessment appointments are scheduled: at baseline, 6 weeks, 6 months, and 12 months. Assessment appointments can occur on the same day as treatment appointments.

##### Baseline Assessment

During the baseline assessment, the vulva will be examined for LS, and vulvoscopy will be performed to assess for signs of vulvar malignancy. A 3-4 mm vulvar biopsy under a local anesthetic will be performed to confirm the diagnosis of LS for participants without prior biopsy-proven evidence. The investigator scores the patients’ LS by using the clinical scoring system, and the participant completes the Visual Analogue Scale, the Australian Pelvic Floor Questionnaire, and the Dermatology Life Quality Index. Deidentified clinical photos are taken.

##### Assessments at 6 Weeks, 6 Months, and 12 Months

At each assessment appointment, the investigator performs a vulvoscopy and scores the participant’s LS by using the clinical scoring system. The participant completes the Visual Analogue Scale, the Australian Pelvic Floor questionnaire, and the Dermatology Life Quality Index questionnaire. Deidentified clinical photos are taken at each appointment. Additionally, participants are asked if they experienced symptoms requiring the use of topical steroids. The completed documents are placed in the patient’s folder and remain onsite in a locked cabinet inaccessible to clinical investigators. Documentation will be accessible by a nonclinical research assistant who will be responsible for entering the collected data into the trial’s SPSS database for statistical analysis. At the final assessment consultation, participants are discharged to the care of their referring physician. At the conclusion of data analysis, the participant and the investigators will be informed about the treatment allocations during the study. Participants will receive a letter about the research findings.

### Outcome Measures

#### Primary Outcome Measure

The primary outcome of this study was to compare the efficacy of PRP and saline injections by evaluating the change in the LS clinical score across groups at 3 timepoints: 6 weeks, 6 months, and 12 months (Table 1). The clinical scoring system for LS is an objective, validated, physician-administered assessment measuring the severity of vulvar LS [18]. Six clinical characteristics are graded on a 3-point Likert scale with grade 0 as normal, grade 1 as moderate changes, and grade 2 as severe changes. The clinical characteristics are erosion, hyperkeratosis, fissures, agglutination, stenosis, and atrophy. Scores range from a minimum of 0 to a maximum of 12; a score ≥4 identifies LS with a probability of >90% in conjunction with the participant experiencing intractable symptoms such as pruritus, dyspareunia, soreness, or burning [22].

**Table 1:** Clinical scoring system for vulvar lichen sclerosus.

Clinical characteristic	Grade 1 (moderate)	Grade 2 (severe)
Erosion	1-2 small erosions, almost not macroscopically visible	Macroscopically visible and/or >2 or confluent lesions
Hyperkeratosis	≤10% vulva and perineum affected	>10% vulva and perineum affected
Fissure	Rhagades affecting the posterior introitus	Generalized vulvar rhagades
Agglutination	Partially affecting preputium clitoridis and labial minora	Complete agglutination of both labia minora or majora
Stenosis	Narrowing of introitus, which could still be passed by 2 fingers	Narrowing, which could be passed by less than 2 fingers
Atrophy	Shrinking of labia and clitoris	Labia minor and clitoris were no longer visible

#### Secondary Outcome Measures

Secondary outcome measures will compare changes in the subjective assessment scores over time within each group and between groups. Participants will complete the Australian Pelvic Floor Questionnaire and Dermatology Life Quality Index at each assessment appointment to evaluate changes in the subjective disease burden. The Australian Pelvic Floor Questionnaire is a validated self-administered questionnaire assessing pelvic floor symptoms, including bladder, bowel, prolapse, and sexual function symptoms, as well as symptom severity and impact on quality of life [23]. The Dermatology Life Quality Index is a validated tool assessing quality of life impairment for people experiencing inflammatory skin conditions, and a change in the score of at least 4 points is considered clinically significant [24]. A Visual Analogue Scale will be presented to participants at each assessment to record the severity of their symptoms. Participants will be asked to indicate their level of distress in relation to 2 symptoms: pruritus and dyspareunia.

The additional secondary outcomes are safety and adverse events of receiving the treatment. Autologous PRP has an advantageous safety profile due to the utilization of the patient’s own blood, which minimizes the risk of adverse events or autoimmune reactions [25]. In a pilot study undertaken by the principal investigator, all participants (n=28) reported minimal to mild pain in the 24 hours following the procedure [20]. There were no reported cases of infection, bleeding, hematoma, or other adverse effects. Adverse events will be monitored immediately after receiving the treatment, and at each assessment appointment, participants will be asked if they experienced any adverse events in the preceding period. Any adverse effects will be recorded and reported to the ethics committee.

#### Sample Size and Power Calculations

Sample size calculations are based on the pilot study on 28 patients [20], in which 80% of the participants treated with PRP experienced an improvement in their symptoms. Assuming a difference of 30% between PRP and normal saline at 12 months, an α risk of 5%, and a power of 80%, a sample size of 31 participants for each group is required. Assuming that 10% of the participants withdraw or are lost to follow-up, a sample size of 34 participants for each group is required or a sample size of 68. The pilot study used subjective symptom improvement as its primary outcome, whereas this trial’s primary outcome is the LS clinical score, an objective physician-rated measure. Although these outcomes differ, symptom improvement in the pilot study was used as a surrogate to estimate a conservative effect size for this study, given the lack of prior data on LS clinical score changes with PRP. This study will also evaluate secondary outcomes, including subjective symptom scores, allowing for comparisons between subjective and objective measures.

### Statistical Analysis

All analyses will be conducted using SPSS software (IBM Corp). We will perform analyses using the intention-to-treat principle. Outcomes will be compared within groups over time and between groups at baseline, 6 weeks, 6 months, and 12 months. Baseline characteristics will be summarized using descriptive statistics, and a comparison of the 2 treatment arms will be assessed by 1-way analysis of variance or 1-sided *t* test for continuous variables or nonparametric alternatives if data are not normally distributed. The Shapiro-Wilk test will be used to assess if data are normally distributed. For all tests, a significant level of α=5% is applied. The analysis of changes compared to the baseline will test the null hypothesis that there is no difference between the 2 timepoints. Further analyses between the groups will test the null hypothesis that there are no differences between the 2 groups at one timepoint. Missing value analysis will be performed, and missing values will be imputed using expectation maximization. Analyses will be conducted in line with the intention-to-treat principle, which will be compared to per-protocol analyses.

## Results

Recruitment for the trial commenced in May 2020 and concluded in November 2023. The study is closed as of September 2024 due to slow recruitment. A total of 34 participants were recruited. Data analysis is being performed in 2025, and the study results are expected to be published in late 2025.

## Discussion

### Principal Findings

PRP has emerged as a novel treatment for the management of a variety of dermatological conditions, including LS, because of its regenerative properties and potential to alleviate symptom burden [14]. To date, a number of studies have been conducted investigating PRP as a treatment for LS; however, these have been either case series or uncontrolled studies or studies that used multiple interventions such as both PRP and adipose-derived stem cells, limiting the ability to differentiate the efficacy of individual interventions [13]. Franic et al [21] highlighted significant symptom improvement in a premenopausal woman treated with PRP, underscoring its potential as a therapeutic alternative in cases resistant to conventional treatment methods.

Despite these limitations, these studies have reported encouraging results in terms of symptom relief for patients with LS, reducing itching, pain, and discomfort [26] but lack adequate power, standardized protocols, and comparison to a control. Other gynecological studies have reported a beneficial effect of PRP in patients with stress urinary incontinence, pelvic organ prolapse, genitourinary syndrome of menopause, vaginal fistula, and LS; however, these studies have the same limitations as described above [20,27].

This study was designed as a randomized controlled trial to fill the gap in existing literature about the efficacy of PRP for the treatment of vulvar LS. It aims to provide other researchers in this field with a standardized protocol, including methods of preparation and injection of PRP and the use of validated outcome measures assessing both the clinical signs of disease and subjective effects of the disease on the quality of life.

### Limitations

As a study protocol, there are several anticipated limitations to consider. This trial is being conducted at a single private clinical center, which may limit the generalizability of findings to other settings, particularly public hospitals or international populations. Although the study is powered to detect a significant difference between groups, the modest sample size may not capture rare adverse events or allow detailed subgroup analysis. Additionally, although outcome assessors and participants are blinded, the treating investigator must remain unblinded for procedural reasons, introducing a potential risk of bias. Lastly, although validated tools such as the clinical scoring system for LS and quality of life measures are employed, patient-reported outcomes are inherently subjective and may be influenced by expectations, even in a placebo-controlled setting.

### Comparison With Prior Work

Previous investigations [20,21] into the use of PRP for vulvar LS have largely consisted of case reports, small cohort studies, and pilot trials, many of which lacked standardization in PRP preparation and outcome measurement. These earlier studies often demonstrated promising symptomatic improvements but were limited by heterogeneity in design, short follow-up durations, and absence of control groups. This protocol addresses several of these gaps by incorporating a parallel-group, double-blind, randomized controlled trial design with standardized PRP preparation, objective clinical scoring, and validated patient-reported outcome measures. By using a clearly defined placebo (saline) control and ensuring long-term follow-up, this study aims to provide robust evidence to inform future clinical practice.

### Conclusions

This protocol outlines a rigorous, placebo-controlled, double-blind randomized controlled trial designed to assess the safety and efficacy of autologous PRP injections for the treatment of vulvar LS. By employing standardized preparation methods, validated clinical and quality of life outcome measures, and long-term follow-up, this study aims to address the methodological limitations of prior research. The findings from this trial have the potential to inform clinical practice by providing high-quality evidence on the role of PRP as an alternative or adjunctive therapy in patients who are unresponsive to standard topical steroid treatment.

## Appendix

Multimedia Appendix 1 Participant information sheet and consent form.

Multimedia Appendix 2 Protocol for platelet-rich plasma preparation and saline preparation.

Multimedia Appendix 3 Participant appointment schedule.

Multimedia Appendix 4 Demographic data collection sheet.

Multimedia Appendix 5 Visual Analogue Scale.

Multimedia Appendix 6 Australian Pelvic Floor Questionnaire.

Multimedia Appendix 7 Dermatology Life Quality Index.

Multimedia Appendix 8 Study recording sheet.

Multimedia Appendix 9 Participant flow diagram.

Multimedia Appendix 10 CONSORT (Consolidated Standards of Reporting Trials) checklist.

Multimedia Appendix 11 SPIRIT (Standard Protocol Items: Recommendations for Interventional Trials) checklist.

## Abbreviations

CONSORT: Consolidated Standards of Reporting Trials
LS: lichen sclerosus
PRP: platelet-rich plasma
