# Supplementary material for: Platelet-Rich Plasma Versus Saline for the Treatment of Vulvar Lichen Sclerosus: Protocol for a Randomized Controlled Trial
Source: JMIR Res Protoc. 2025 Sep 3;14:e68871. doi: 10.2196/68871 (PMC12444214; doi:10.2196/68871)
Supplement: Multimedia Appendix 1 [file resprot_v14i1e68871_app1.docx]

## Multimedia Appendix 1: Participant information sheet and consent form

This study was developed for assessing a treatment option for women with lichen sclerosus. You have been approached about this study due to your diagnosis of **vulvar lichen sclerosus**.

The recommended treatment for lichen sclerosus is topical high dose steroid therapy on the vulva. However, some women, such as yourself, do not tolerate or are unable to use this treatment. Therefore, this study is designed to assess an experimental treatment for vulvar lichen sclerosus.

The experimental treatment introduced in this study is **Platelet rich plasma (PRP).** This treatment is used or being tested in other areas of medicine, including dentistry, for musculoskeletal injury, and cosmetic procedures. It involves treating your vulvar lichen sclerosus with your own blood material.

The mechanism of action for PRP is to stimulate cell regeneration and promote tissue development through the concentrated growth factor recruitment in the area of injection.

When a blood sample is collected from your arm and spun at a fast speed, the blood separates into a dark red section and a pale yellow straw-coloured section. It is this straw-coloured section that is known as plasma. To make PRP, your blood is collected into a specially designed tube. Once spun, the straw-coloured section is collected into a syringe and injected into the part of your body needing treatment; in this case, your vulva.

PRP can cause some mild pain, as it involves using a needle to collect your blood to make the PRP and a smaller needle to inject under the vulvar skin. The investigators will explain these treatments to you and address any questions you may have.

**Invitation to participate:**

We invite you to participate in this research project. Regardless of your decision, your medical care/relationship with your gynaecologist will not be affected.

**Aims of the project:**

The aim of this project is to determine whether the platelet-rich plasma is useful for the treatment of vulvar lichen sclerosus.

**Summary of procedures:**

Your invitation to this study means that a gynaecologist has already reviewed and diagnosed you with vulvar lichen sclerosus and you have not tolerated or are unable to use topical high dose steroid therapy.

This is a randomised control trial. You may receive the experimental treatment injections, which is PRP, or placebo injections, which is normal saline. A random process will decide which treatment you will receive. There will be 68 participants to be enrolled in this study. You will not be aware of the treatment until after the trial, which is after completion of the 12-month follow-up. The study doctor will know your treatment allocation so that you receive the assigned treatment. The assessing doctor will not know your treatment allocation to ensure independent assessment. The investigators will know the results of this study after the completion of the trial.

If you choose to participate in this study, your participation will include the following:

1. **An initial assessment consultation**.
   - This will involve completing a questionnaire and undergoing a vulvar examination, including a vulvoscopy to assess and score the vulvar lichen sclerosus. A vulvoscopy is a magnified view of the outside of the vagina by using a telescope-like device, called a colposcope. You will have a small (3-4mm) vulvar biopsy under local anaesthetic to confirm lichen sclerosus if this has not already been performed.
   - De-identified clinical photos will be taken. This consultation takes 45-60 minutes.
2. **Two follow-up assessment consultations and two treatments**
   - The assessment consultations will involve collecting the same information as your initial assessment consultation – this will be the questionnaire and vulvar examination. This allows the investigators to compare information before and after your study treatment.
   - The first treatment may occur on the same day as your first assessment.
   - The second assessment and treatment is 6 weeks after your first treatment.
   - The third assessment is 6 months after your first treatment.
   - The final assessment is 12 months after your first treatment.
   - De-identified clinical photos will be taken. These consultations take 30-45 minutes.

There will be 2 specialists involved in your care. The first specialist is the study doctor, who will administer the study treatment, which could be experimental (PRP) or placebo (normal saline). The second specialist will be the assessing doctor at your follow-ups, who will perform the assessment.

There will be no medical expenses involved at any of these consultations or treatments. The dates for these appointments will be given to you at the initial consultation appointment.

The assessment consultations involve collecting information about your diagnosis and quality of life, to determine whether the treatment has had any effect on your condition. Given your condition is of a gynaecological nature, this will involve personal questions, such as your sexual experiences. Please discuss this with the study or assessing doctor if you have any concerns.

The treatment involves drawing 8mls of your own blood for processing to 4mls of PRP. You will be requested to lie on a gynaecological examination chair. The Principal Investigator will apply topical anaesthetic cream to your vulva ten minutes prior to injecting the randomised study treatment, PRP or normal saline, underneath your vulva that is affected by lichen sclerosus by using a small (30-gauge) needle. Please avoid sexual intercourse for 72 hours after treatment.

**Commitments:**

If you choose to be involved in this study, you will be asked to attend the clinic four times over a 6-month period. These times will be negotiated with you during your initial assessment consultation and you will be given an appointment card with the dates.

**Benefits:**

During this study, your symptoms may remain the same, worsen, or improve. There is no guarantee that your symptoms will alleviate with the experimental treatment.

**What will happen to me at the end of the study?**

After your last assessment consultation, you will have finished your role in this study. You may remain a patient of the study doctor and receive ongoing care from him/her in a normal medical relationship.

**Risks:**

There is a risk that you will not feel better after receiving the full treatment. There is a risk that the treatment may cause slight discomfort for you. There is also a risk that you may react in an unexpected way to the treatment. This may involve hypersensitivity (allergic reaction), infection, and bleeding. You are encouraged to discuss your concerns with the clinician if it does occur. Treatment will be offered for any adverse reaction. You will not incur any expenses should you require treatment for the adverse reaction.

**Compensation:**

You will not be charged for any of your treatment or assessments related to this study. However, you will be required to arrange your own traveling to your appointments. Travel vouchers will be available upon request.

If this study does not suit you emotionally or physically, you may withdraw from this study and your care will not be affected in any way. By participating in this study, you do not give up your legal rights.

If, as a result of your participation in this study, you become ill or are injured, immediately advise your study doctor of your condition. In the first instance, your study doctor will evaluate your condition and then discuss treatment with both you and your regular treating doctor.

Since you are participating in a non-sponsored study, any question about compensation must initially be directed to your study doctor who should advise their insurer of the matter.

**It is the recommendation of the independent ethics committee responsible for the review of this study that you seek independent legal advice.**

**Confidentiality:**

All records containing personal information will remain confidential and no information which could lead to your identification will be released, except as required by law.

Your details will be recorded and stored in an electronic medical record and will not be destroyed. The data from this study will be coded and kept in an excel spreadsheet that is password protected. This spreadsheet will be located at the study site.

**Publication:**

The information collected from you during this study will be used to work out whether the study treatment is better or worse than the current treatments offered for your condition. The overall results of this study will be shared with the medical profession through scientific publications, presentations and meetings. This information will not contain any information that may reveal your identification.

**Withdrawal:**

You may choose to withdraw from this study at any time. If you wish to withdraw, you can choose to remove any information collected on you. You can also choose to allow your data to remain in the study. Written consent will be obtained for the ongoing use of your data. Withdrawal from this study will not have any impact on your ongoing medical care with your doctor.

**Outcomes:**

If you wish, you can ask us to send you any publications we make on this project.

**Contact:**

If you would like more information about this project, please contact Dr Fariba Behnia-Willison at FBW Gynaecology Plus on 08 8297 2823.

The Bellberry Human Research Ethics Committee has reviewed and approved this study in accordance with the National Statement on Ethical Conduct in Human Research (2007) incorporating all updates. This Statement has been developed to protect the interests of people who agree to participate in human research studies. Should you wish to discuss the study or view a copy of the Complaint procedure with someone not directly involved, particularly in relation to matters concerning policies, information or complaints about the conduct of the study or your rights as a participant, you may contact the Committee chair, Bellberry Human Research Ethics Committee on 08 8361 3222.

**Consent for Participation in this Study**

I ……………………………………………………the undersigned hereby voluntarily consent to my involvement in the research project titled: **The role of regenerative medicine for treatment of Lichen Sclerosus: A randomised control trial of Platelet-Rich Plasma (PRP) versus Normal Saline**.

I acknowledge that the nature, purpose and risks of the research project and alternatives to participation have been fully explained to my satisfaction by Dr ……………………………………………………

Specifically, the details of the procedure proposed and the anticipated length of time it will take, the frequency with which the procedure will be performed and an indication of any discomfort that may be expected have been explained to me.

I freely agree to participate in this research project according to the conditions in the Participant Information Sheet which I confirm has been provided to me.

I understand that my involvement in this study may not be of any direct benefit to me.

I have been given the opportunity to have a member of my family or another person present while the study is explained to me.

I have been told that no information regarding my medical history will be divulged to understand third parties and the result of any tests involving me will not be published so as to reveal my identity.

I understand that I am free to withdraw from the study at any stage without prejudice to future treatment. If I decide to withdraw from the study, I agree that the information collected about me up to this point when I withdraw may continue to be processed or excluded altogether.

I am 18 years of age or over.

I consent to my treating doctor/s being notified of my participation in this study and of any clinically relevant information noted by the trial doctor in the conduct of the trial.

I declare that all my questions have been answered to my satisfaction.

I have read, or have had read to me in a language in which I am fluent, and I understand the Participant Information Sheet, version 4.

| Name of study participant: | |
| --- | --- |
| Signature of study participant: | Date: |

**Declaration by senior researcher*:**

A verbal explanation of the research project, its procedures and risks has been given to the participant and I believe that the participant has understood that explanation.

| Name of senior researcher: | |
| --- | --- |
| Signature of senior researcher: | Date: |

* A senior member of the research team must provide the explanation and provision of information concerning the research project.
