# Supplementary figures and images for: Platelet-Rich Plasma Versus Saline for the Treatment of Vulvar Lichen Sclerosus: Protocol for a Randomized Controlled Trial
Source: JMIR Res Protoc. 2025 Sep 3;14:e68871. doi: 10.2196/68871 (PMC12444214; doi:10.2196/68871)

## Multimedia Appendix 2: Protocol for PRP preparation and saline preparation


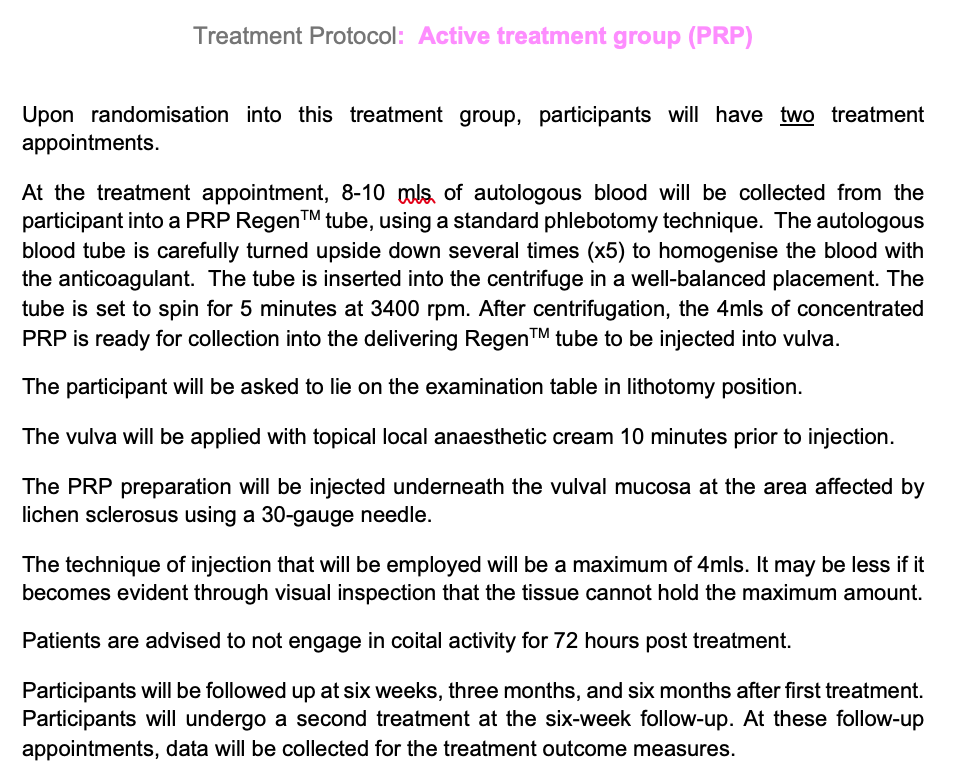


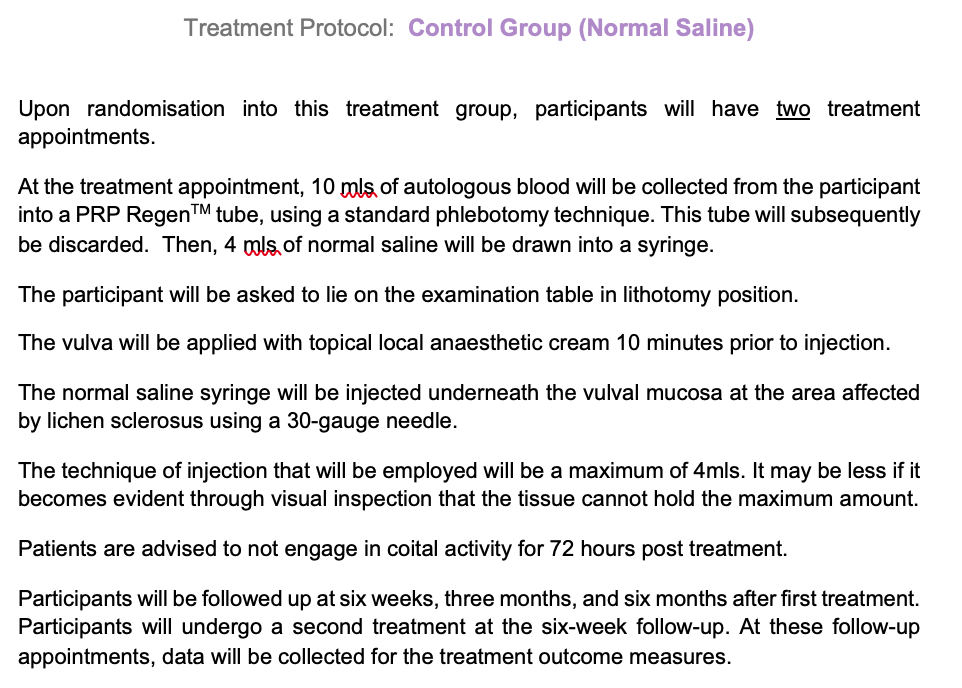

Supplement: Multimedia Appendix 2 [file resprot_v14i1e68871_app2.docx]

## Multimedia Appendix 3: Participant appointment schedule


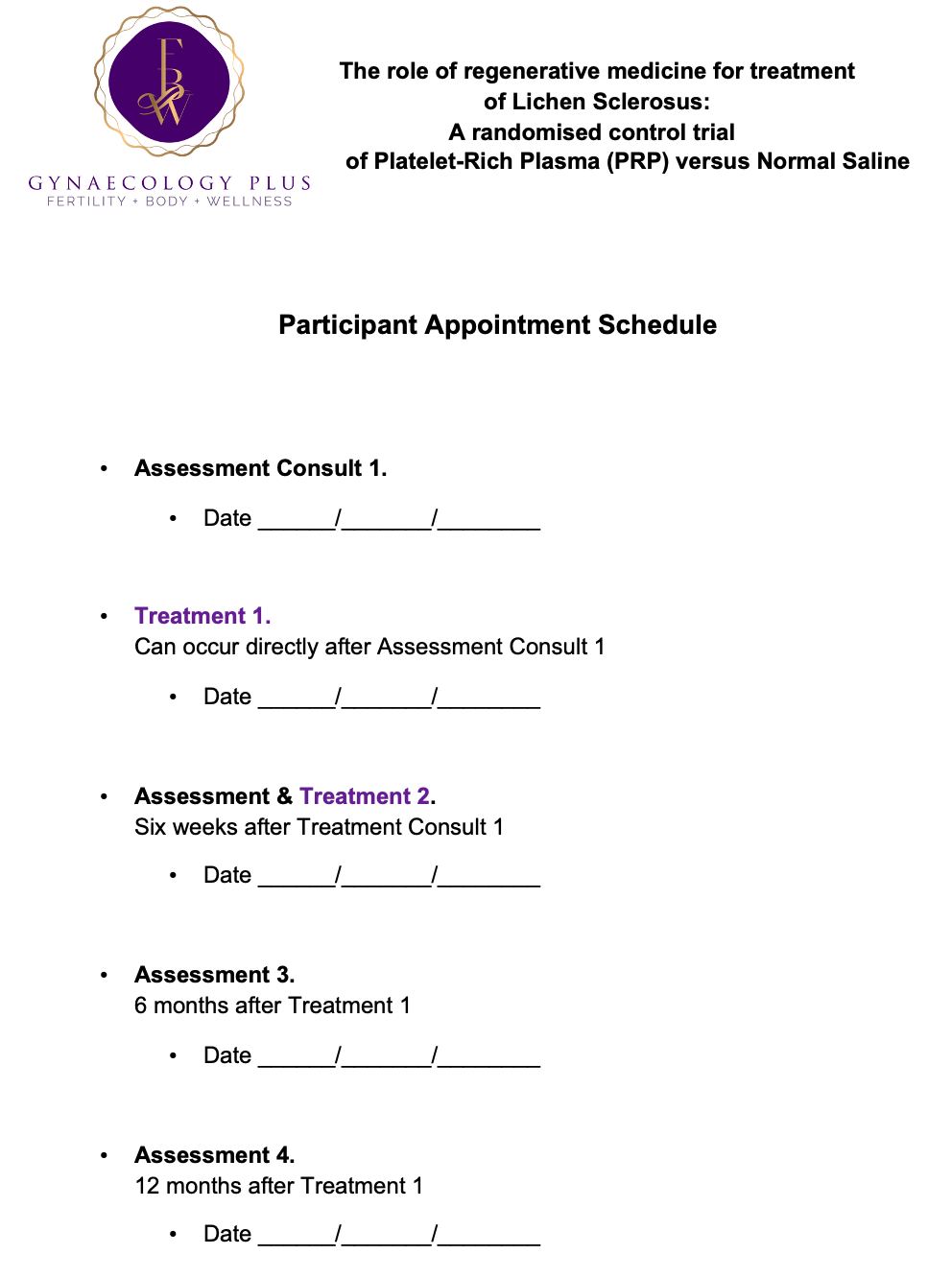

Supplement: Multimedia Appendix 3 [file resprot_v14i1e68871_app3.docx]

## Multimedia Appendix 4: Demographic data collection sheet


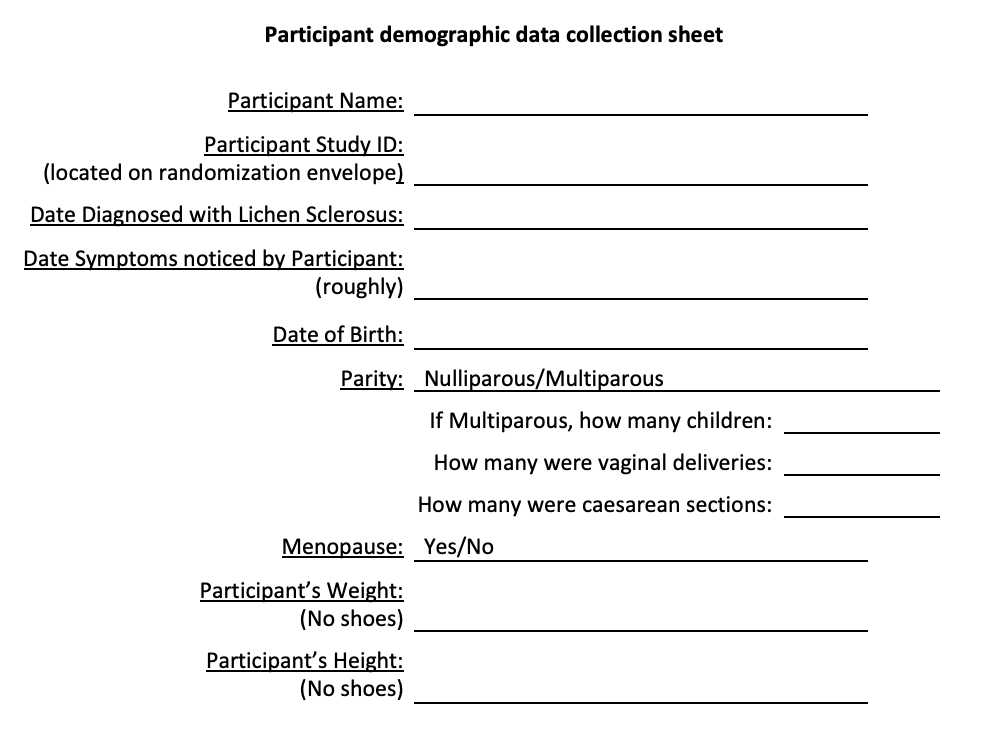

Supplement: Multimedia Appendix 4 [file resprot_v14i1e68871_app4.docx]

## Multimedia Appendix 6: Australian Pelvic Floor Questionnaire


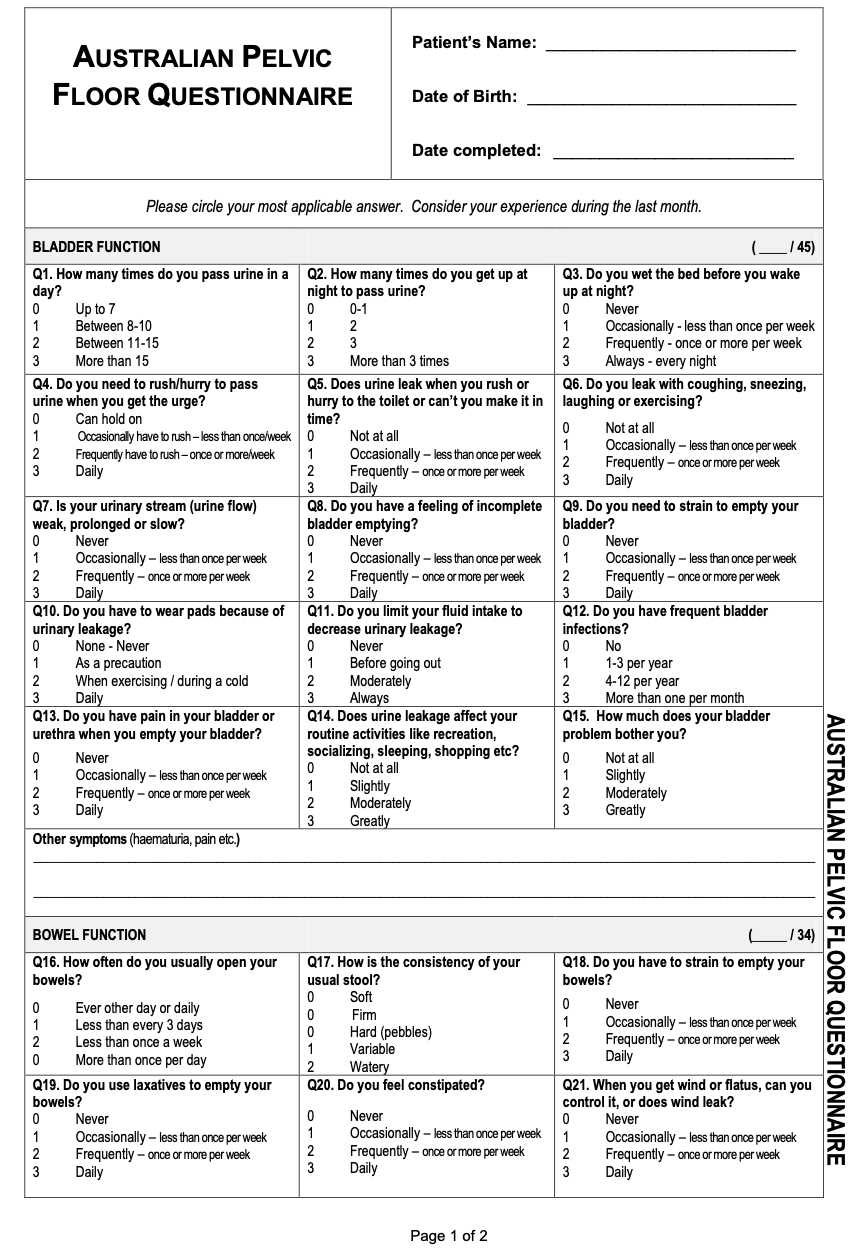


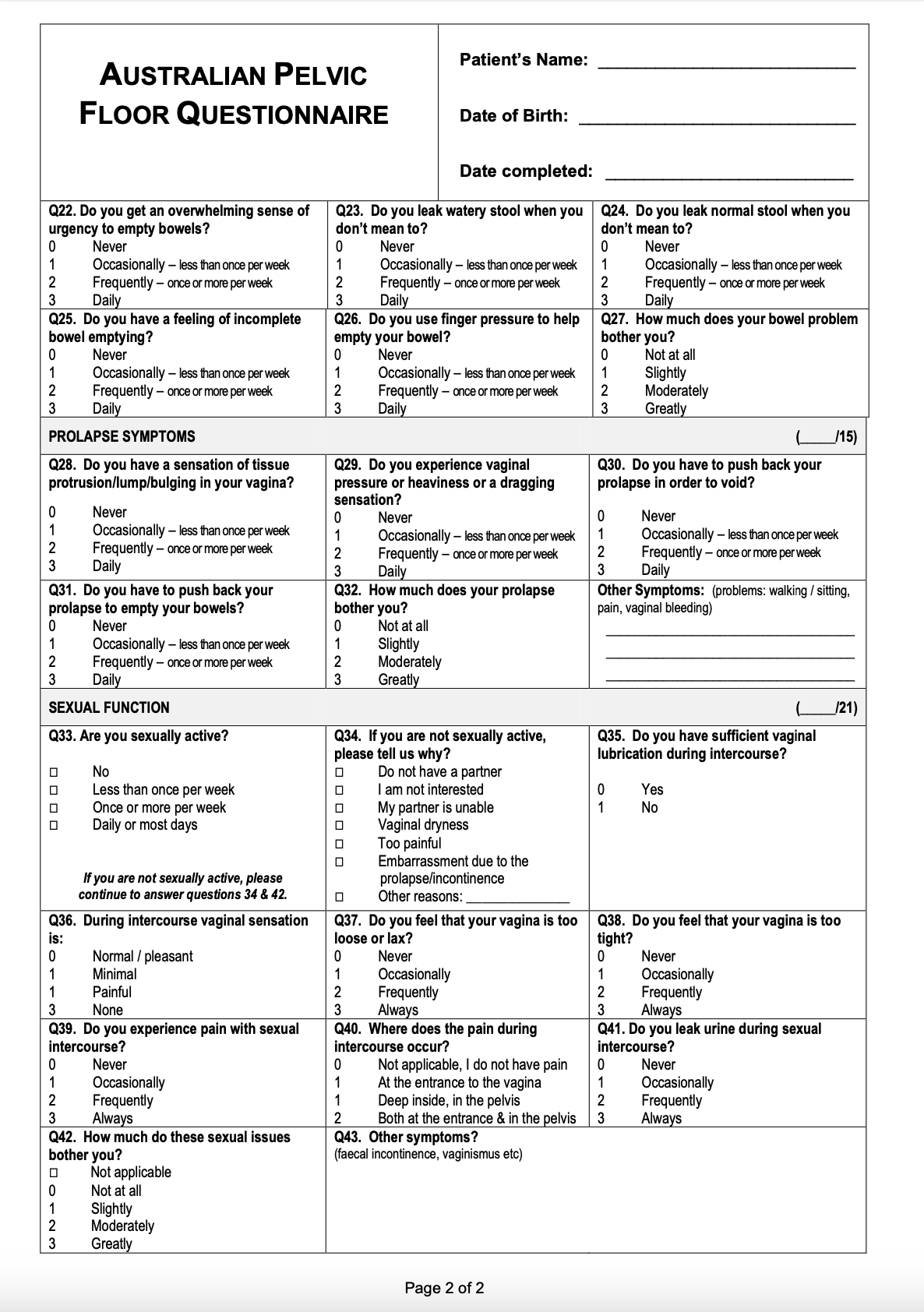

Supplement: Multimedia Appendix 6 [file resprot_v14i1e68871_app6.docx]

## Multimedia Appendix 8: Study recording sheet


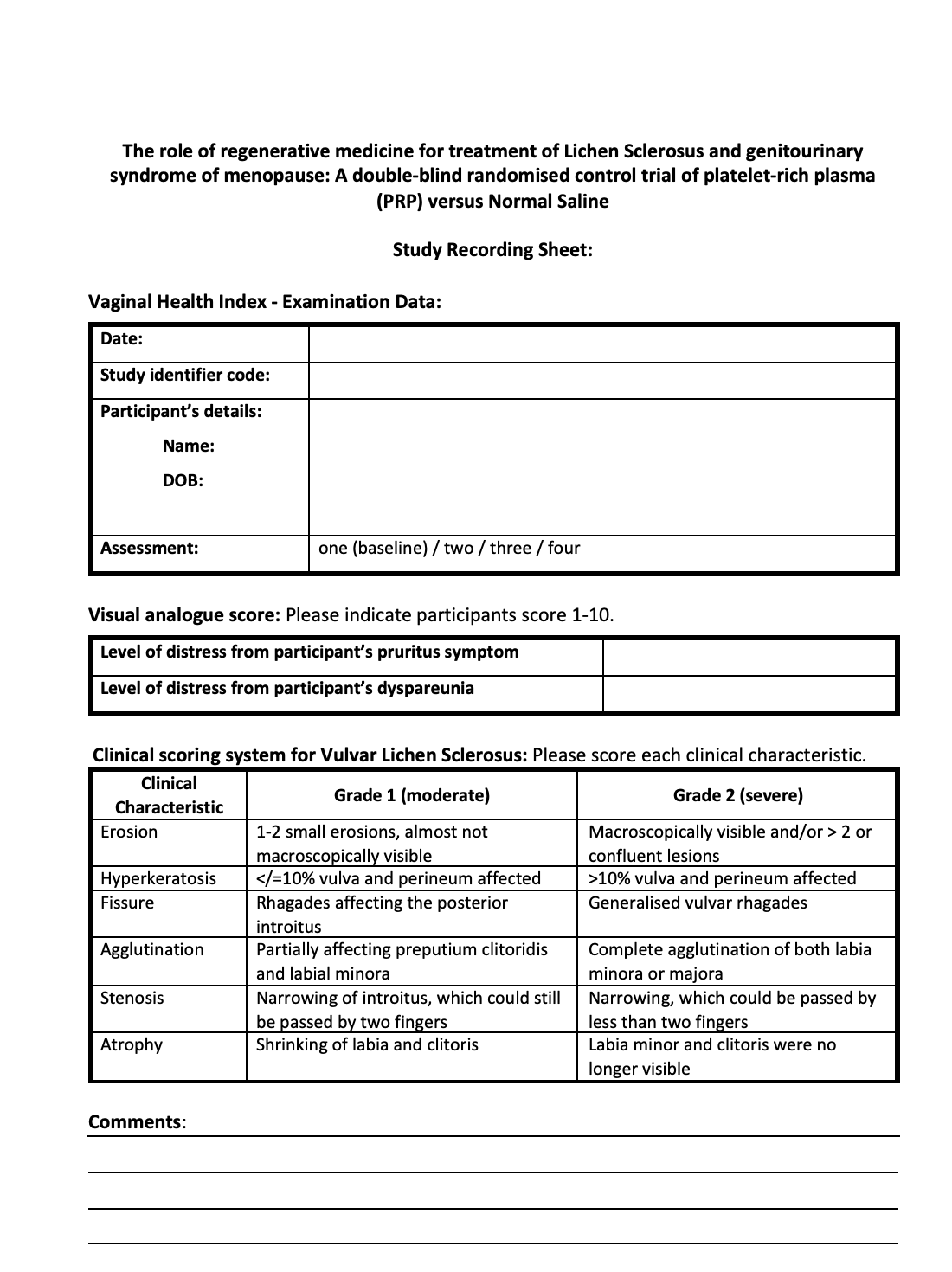

Supplement: Multimedia Appendix 8 [file resprot_v14i1e68871_app8.docx]
