# Supplementary material for: Platelet-Rich Plasma Versus Saline for the Treatment of Vulvar Lichen Sclerosus: Protocol for a Randomized Controlled Trial
Source: JMIR Res Protoc. 2025 Sep 3;14:e68871. doi: 10.2196/68871 (PMC12444214; doi:10.2196/68871)
Supplement: Multimedia Appendix 5 [file resprot_v14i1e68871_app5.docx]

## Multimedia Appendix 5: Visual Analogue Scale
